# Blunt Trauma Neck with Thyroid Cartilage Subluxation with no External Sign

**DOI:** 10.1007/s12070-024-04841-2

**Published:** 2024-07-09

**Authors:** Zeeshan Ahmad, Kriti Singh

**Affiliations:** MLN Medical Collge: Moti Lal Nehru Medical College prayagraj, Uttar Pradesh, India

## Abstract

Trauma to the neck can produce catastrophic results as neck houses plethora of vital structures and is relatively an unprotected anatomical territory. Blunt trauma to the neck, excluding cervical spine injuries, represents only 5% of all neck trauma, but can be very challenging to assess since its presentation is often delayed. Penetrating injuries, on the other hand, are more common, and even when they seem to be only superficial and minor, always need thorough investigation and observation. Primary stabilization of the patient followed by an extensive evaluation needs to be done in all cases of neck trauma. CECT neck and thorax along with fibreoptic laryngoscopy remain the main modalities of diagnosis following a neck trauma. The initial approach to any kind of neck injury, whether penetrating or blunt, is performed according current Advanced Trauma Life Support (ATLS) or European Trauma Course (ETC) protocols, which both use the structured ‘ABCD’ approach. A motor vehicle accident (MVA) remains the most common cause of blunt neck injury, causing direct pressure to the anterior neck from the dashboard, steering wheel or airbag deployment. Direct pressure to the neck is transduced to the trachea and causes compression of the oesophagus against the cervical spine. Laryngotracheal trauma continues to be a rare entity and is the second most common cause of death in patients with head and neck trauma after intracranial injury. Only 0.5% of multiple trauma patients were reported to have injury to the airway at any level. Stabilize the airway first. Immediate surgical airway procedure can be necessary in less familiar circumstances and environments. If possible, define landmarks before the procedure. Defining anatomical zones is useful in penetrating injuries, although these do not guide diagnostic or therapeutic management completely. In unstable patients, elective surgical exploration is recommended instead of extensive diagnostic work-up. Unstable patients still need immediate exploration, whereas all stable patients will first be assessed with clinical examination and CT angiography and fibreoptic laryngoscopy. Thus the take home message is to consider all neck injuries an emergency and proceed with the diagnosis and management without delay.

## Introduction

Trauma to the neck can produce catastrophic results as neck houses plethora of vital structures and is relatively an unprotected anatomical territory. The neck contains several major vessels that lie relatively superficially and are protected only by their fascia and the overlying soft tissues. The same holds true for the aerodigestive tract, which is even less protected by surrounding layers [1, 2].

Blunt trauma to the neck, excluding cervical spine injuries, represents only 5% of all neck trauma, but can be very challenging to assess since its presentation is often delayed. Penetrating injuries, on the other hand, are more common, and even when they seem to be only superficial and minor, always need thorough investigation and observation [3].

## Case Report

**Fig. 1:**
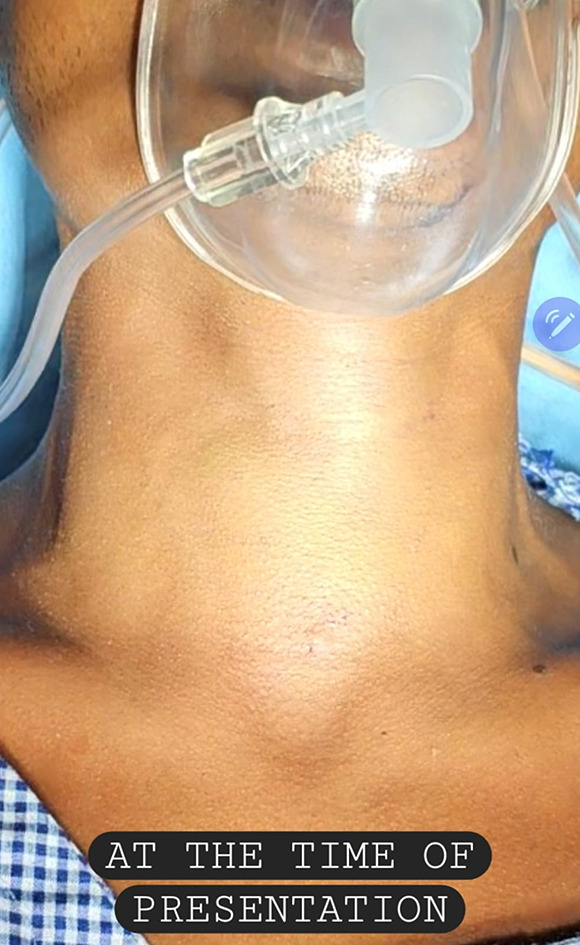
Patient at time of first presentation to hospital

**Fig. 2:**
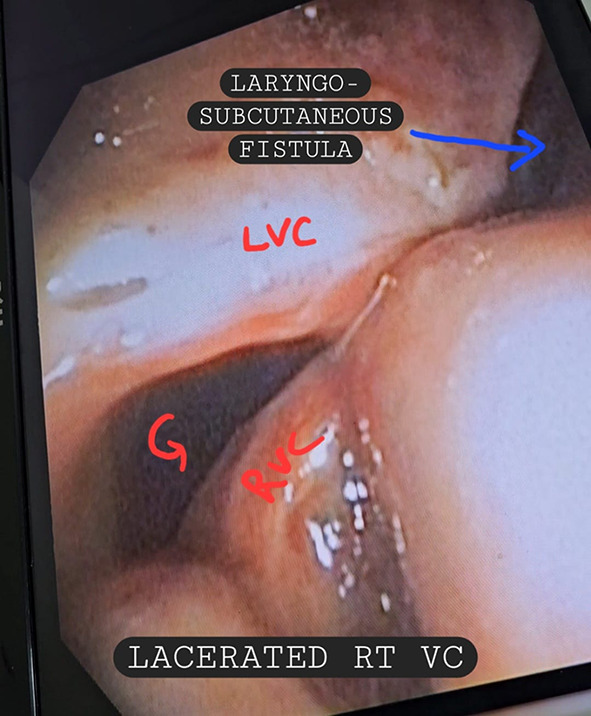
Fibreoptic laryngoscopic picture

**Fig. 3:**
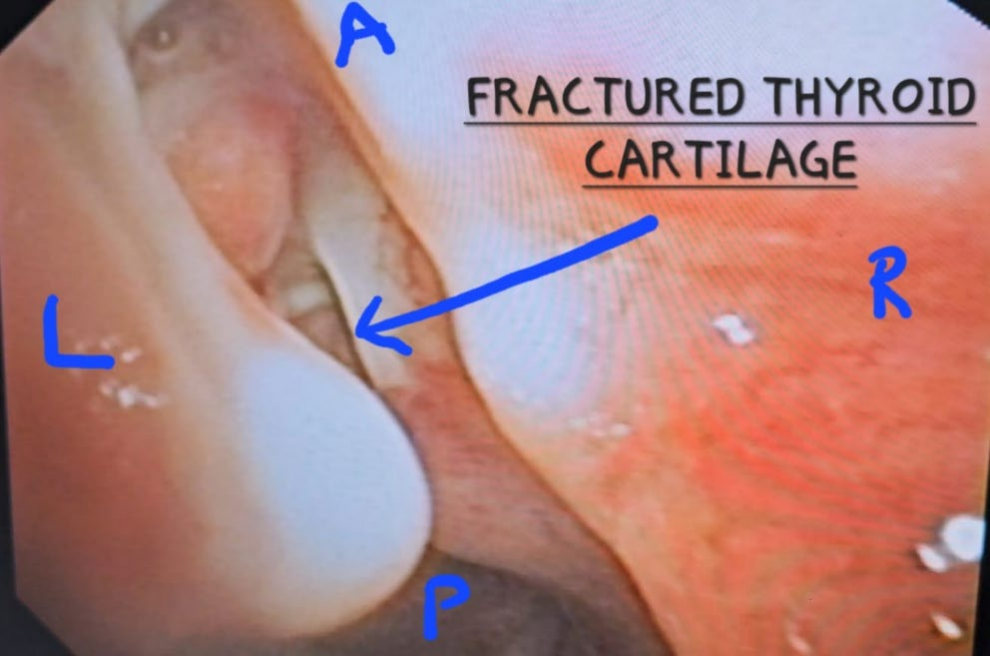
Fibreoptic laryngoscopic picture

We hereby present a case of 26 years old male who presented to the emergency department with complains of hoarseness of voice, hemoptysis, breathlessness, inability to swallow and pain. The patient had history of blunt trauma to the neck by impact of collision on deskboard of a car. There was an immediate episode of hemoptysis after the accident but the patient was vitally stable. He presented to the emergency the next day with lowered saturation and above stated symptoms.The concerned departments which included Department of Emergency Medicine, Department of ENT and HNS, and Department of Pulmonology were paged and an extensive evaluation was done. On our evaluation following were the findings: There was mild bruising on neck with no visible laceration or edema (Fig. 1). On palpation, there was tenderness with subcutaneous crepitations. After stabilizing the patient Fibreoptic laryngoscopy was performed and following findings were recorded: The laryngeal surface of epiglottis showed bruising with laceration at its inferior attachment. Right vocal cord was severely lacerated with no mobility while left vocal cord was mildly lacerated and mobile. Fractured cartilage was seen in anterior larynx (Figs. 2 and 3). Visualized subglottis was normal.

NCCT Neck revealed significant bilateral cervical region post-traumatic emphysema extending into axillary regions and upper part of thoracic wall with evidence of breach along the anterior wall of larynx. There was evidence of air loculi along central spinal canal extending from C2 to C7. Mild air loculi noted around left vertebral artery.

**Fig. 4:**
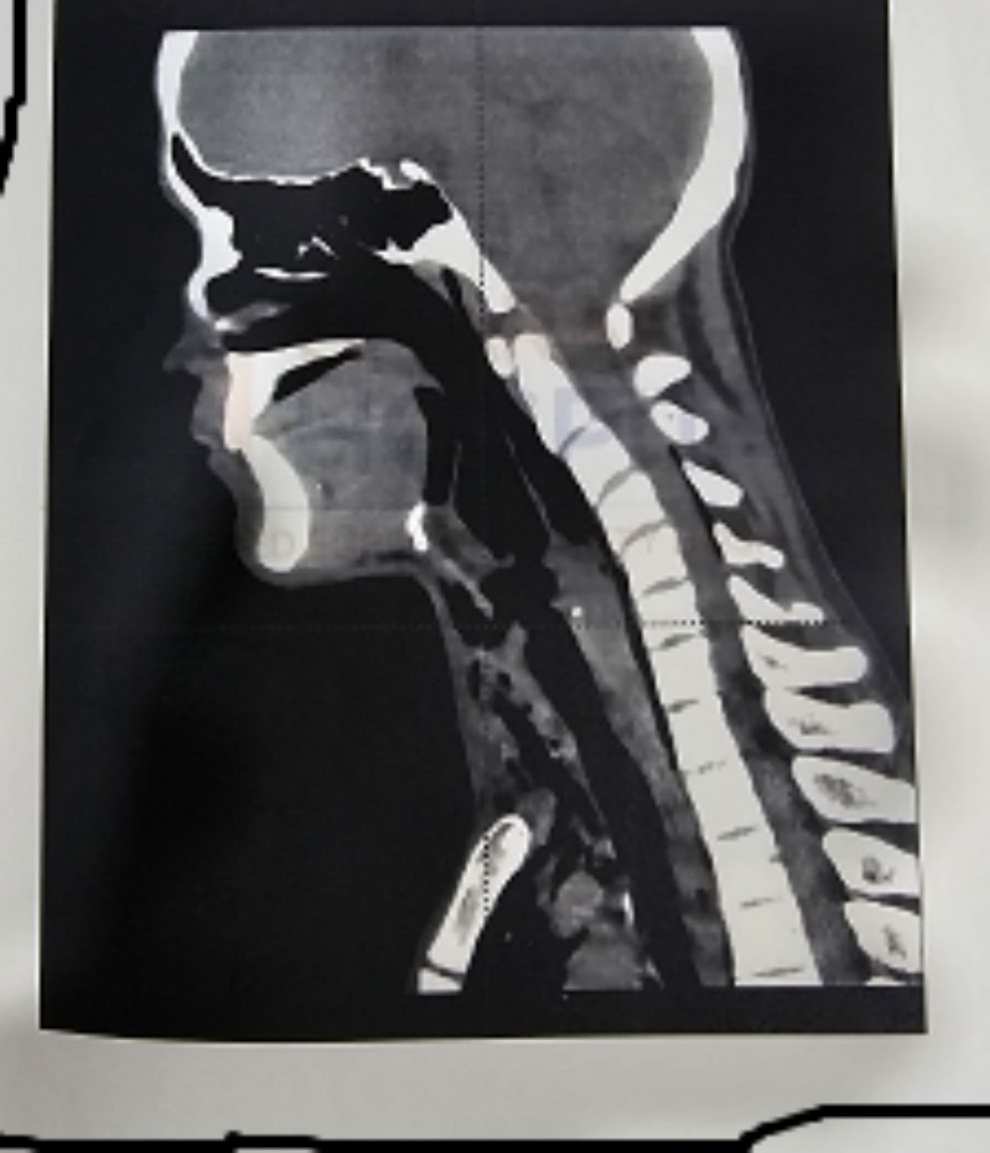
NCCT NECK

**Fig. 5:**
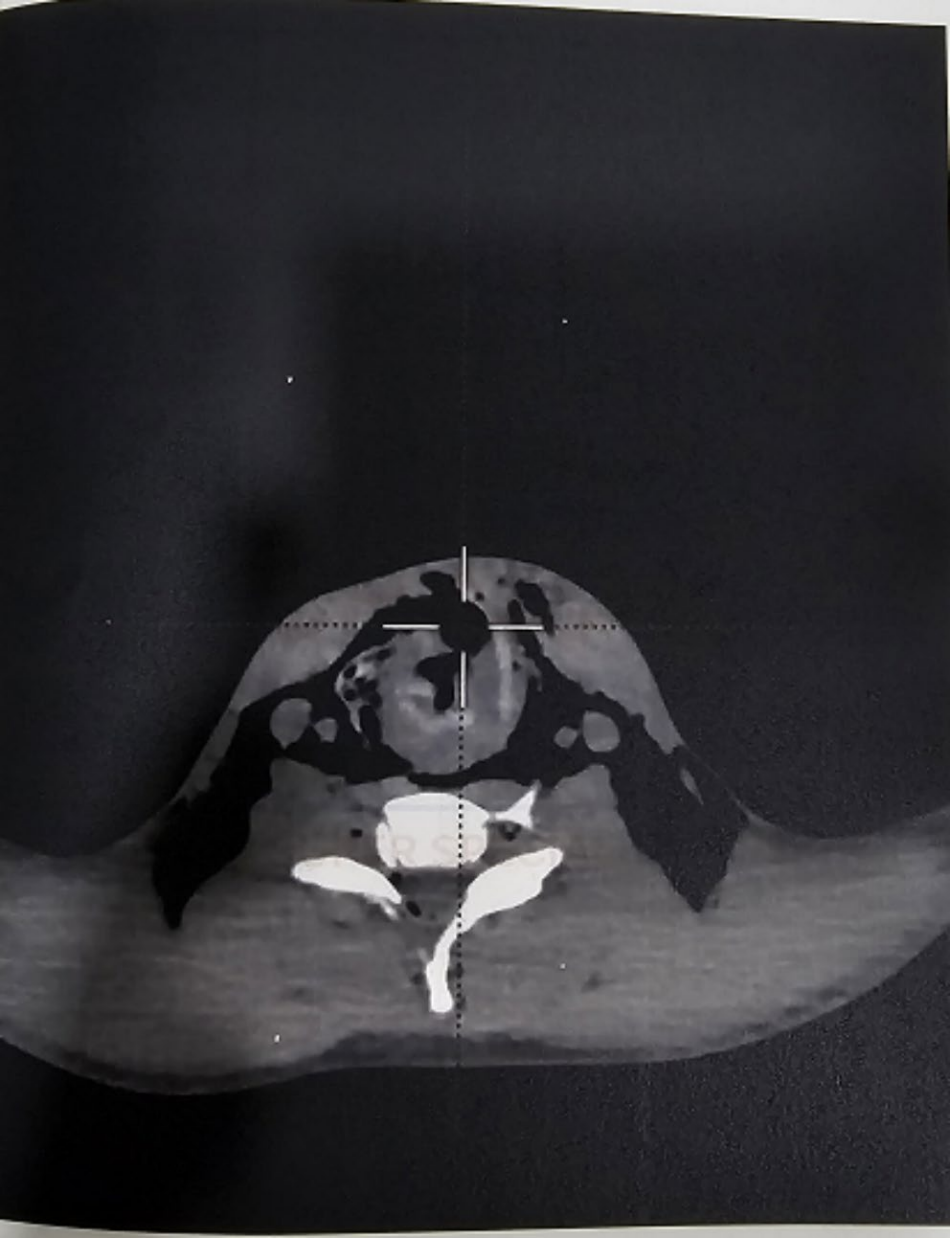
NCCT NECK

**Fig. 6:**
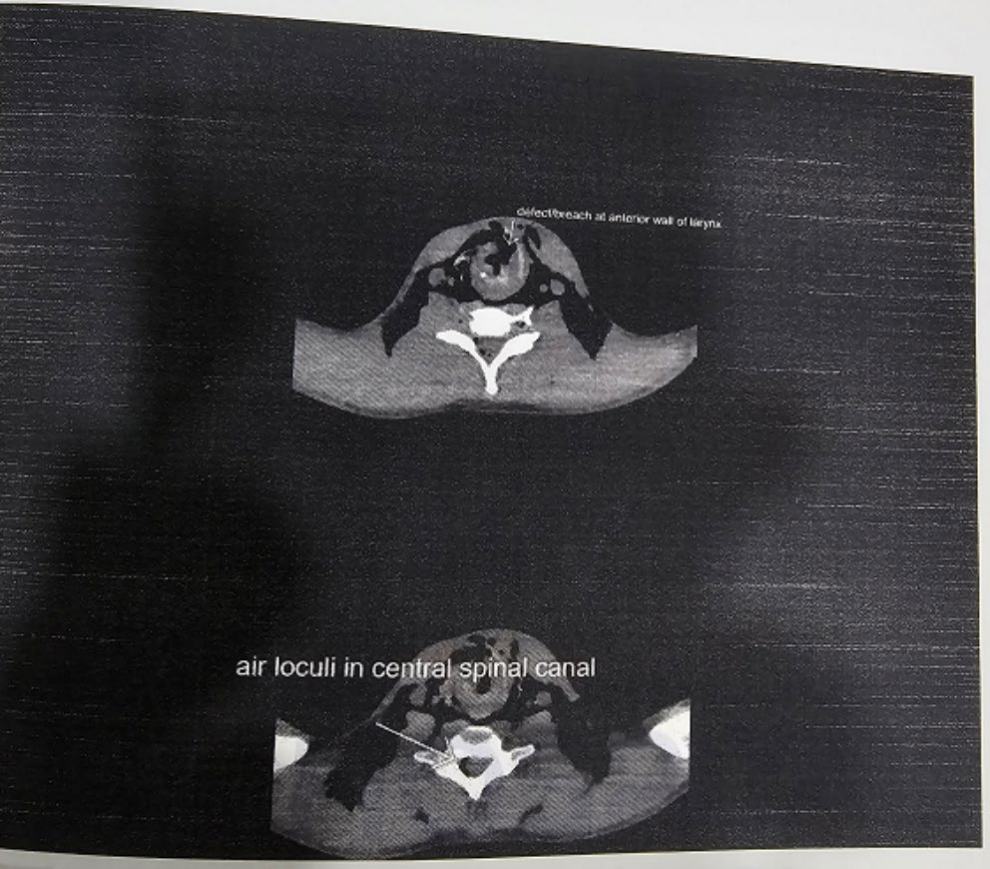
NCCT NECK

CECT Chest revealed mild pneumopericardium with pneumomediastinum. Multiple patchy ground glass radia opacities noted in bilateral lung fields involving all lobes suggestive of pulmonary contusion. Rest findings were same as that of NCCT Neck (Figs. 4, 5 and 6).

The patient was shifted to OT for neck exploration and repair. Midline vertical incision was given and subplatysmal flap was elevated. Right thyroid ala was found fractured and superiorly dislocated near hyoid. Cricoid ring had two fractures.Dissection done and thyroid ala sutured together using prolene.Cricoid fractures sutured together along with approximation of mucosal tears.erocel pack inserted from tracheostomy site and pushed upwards. Flexible laryngoscopy done to ensure complete repair and wound closure done in layers.

## Discussion

**Fig. 7:**
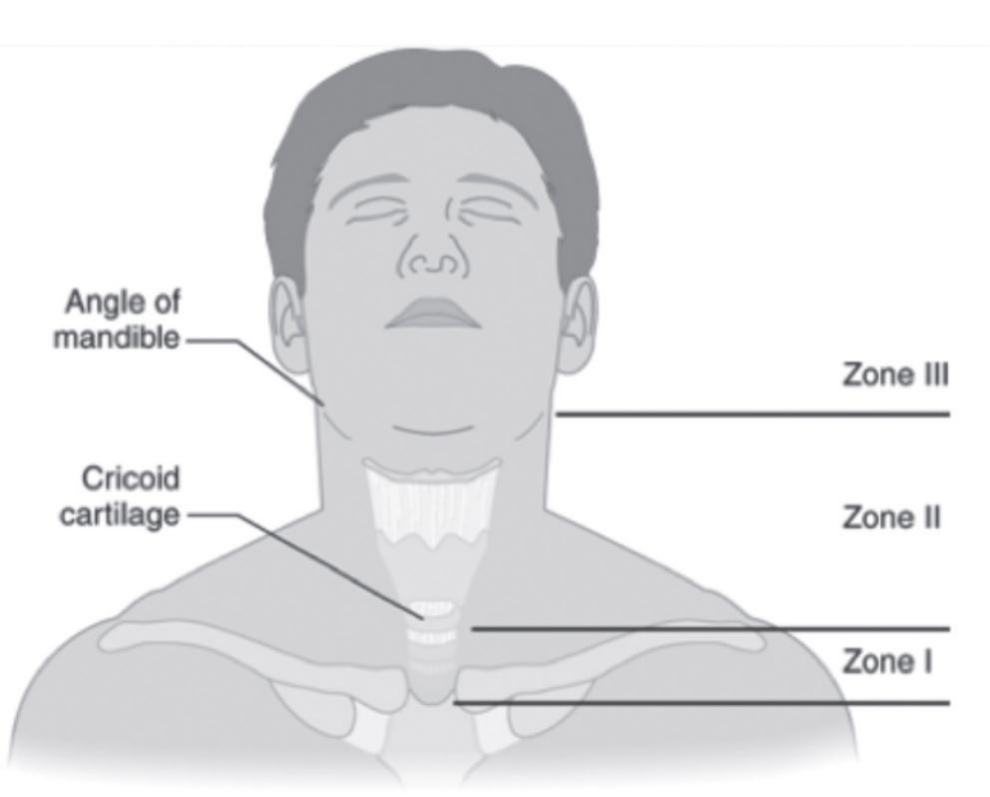
Roon and Christensen classification of anatomical division of neck

Historically, neck has two anatomical divisions - anterior and posterior triangles, divided by the sternocleidomastoid muscle. An alternative approach to anatomical division of neck was presented by Roon and Christensen, who divided the anterior neck into three zones [4, 5] (Fig. 7; Table 1).

**Table 1:** Landmarks and features of the anatomical zones of the neck [5]

Anatomical Zones	Features
Zone 1	-From clavicles to cricoid cartilage - Defines area containing major vital structures - Is considered to be well protected Contains: intrathoracic major vessels subclavian veins and arteries, the proximal vertebral and carotid arteries oesophagus, proximal trachea, larynx brachial plexus, spinal cord superior mediastinum, pleura
Zone 2	-From cricoid cartilage to the angle of the mandible - Defines area containing all vital structures - Is considered to be least protected, very superficial Contains: Carotid arteries, jugular veins, vertebral arteries Trachea, larynx, oesophagus Spinal cord
Zone 3	- From the angle of the mandible to the base of the skull - Very confined, protected area - Difficult to assess and explore Contains: Distal vertebral and carotid arteries Pharynx Spinal cord

**Fig. 8:**
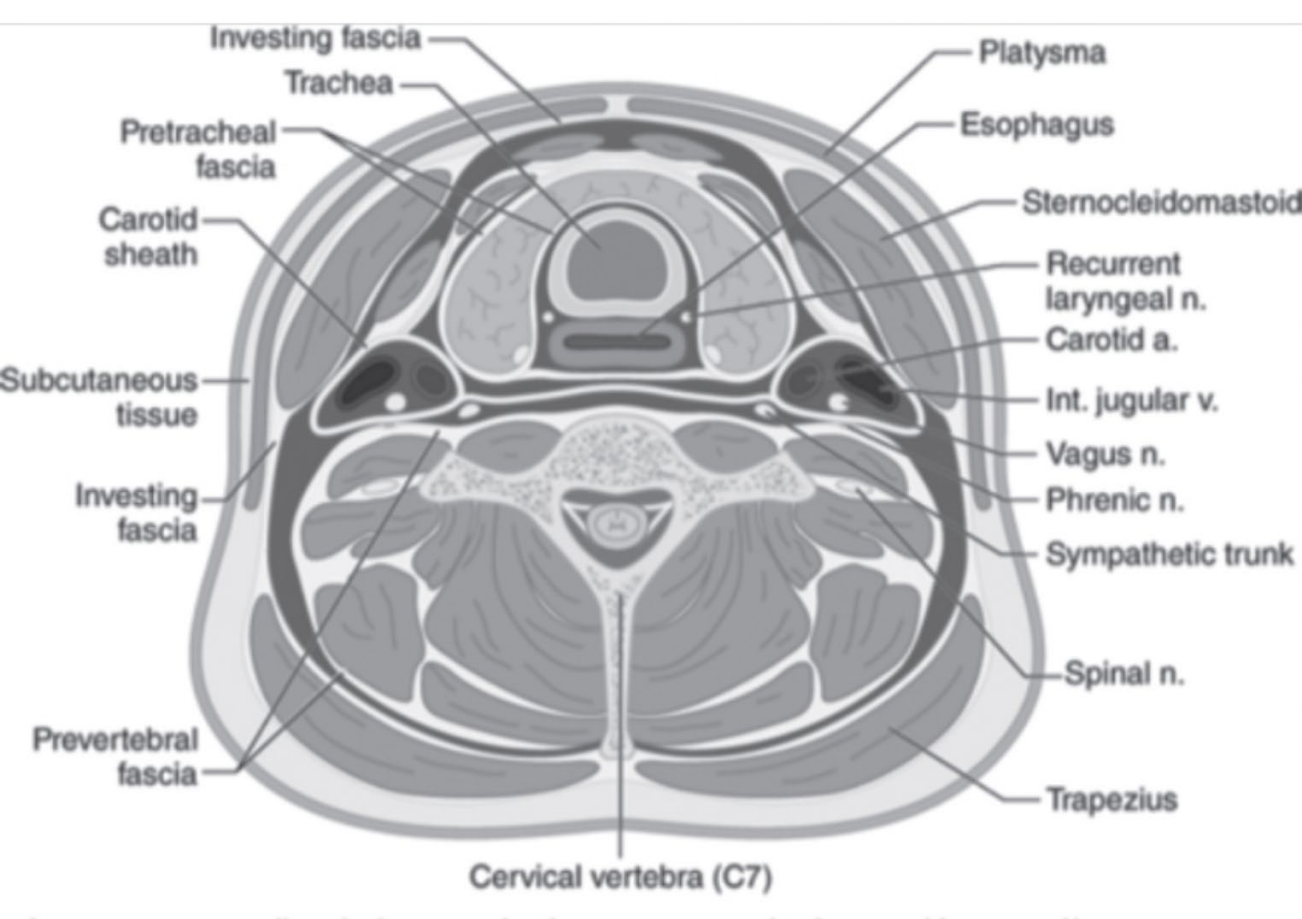
Fascial layers of neck

The neck is protected by several fascial layers, as can be seen in Fig. 8. The superficial fascia surrounds the platysma muscle, covering the anterior and lower parts of the posterior triangle, and is the most superficial structure beneath the skin and soft tissues. It serves as an important anatomical landmark, as a breach of the platysma muscle defines a penetrating injury as deep, mandating thorough investigation and, more often, surgical exploration.

The initial approach to any kind of neck injury, whether penetrating or blunt, is performed according current Advanced Trauma Life Support (ATLS) or European Trauma Course (ETC) protocols, which both use the structured ‘ABCD’ approach [6, 7].

Only 5% of all neck injuries are caused by blunt trauma, and the presentation can range from immediate death and life-threatening airway obstruction to more subtle and insidious signs, making the diagnosis less evident. A motor vehicle accident (MVA) remains the most common cause of blunt neck injury, causing direct pressure to the anterior neck from the dashboard, steering wheel or airbag deployment [3, 8]. Direct pressure to the neck is transduced to the trachea and causes compression of the oesophagus against the cervical spine. Falls, direct blows to the anterior neck during sport or fights, impact from a bicycle handlebar or clothesline injuries are all well described causes of blunt neck trauma. Strangulation by hanging, manual choking or ligature suffocation can cause venous jugular obstruction, intracranial hypertension and unconsciousness, and consequently direct pressure on the trachea and obstruction of the airflow, leading to asphyxiation [9].

Blunt trauma to the oesophagus has a good outcome if the diagnosis is established early, although mortality increases rapidly after 24 h. Symptoms can be very clear (odynophagia, dysphagia, drooling, haematemesis/blood in the gastric tube, subcutaneous emphysema) or very subtle to asymptomatic. Unrevealed oesophageal rupture, however, can lead to a delayed presentation with airway compromise due to haematoma or with mediastinitis, which both carry a poor prognosis.

Blunt trauma to the glottis presents more clearly in most patients. Supraglottic injuries mainly will present with cervical surgical emphysema, palpable cricoid cartilage disruption, hoarseness, dysphagia and/or evolving airway obstruction. Subglottic injuries present more subtly with haemoptysis (or blood in the endotracheal tube) and a persistent air leak after endotracheal intubation [10].

Laryngotracheal trauma continues to be a rare entity and is the second most common cause of death in patients with head and neck trauma after intracranial injury [11]. Only 0.5% of multiple trauma patients were reported to have injury to the airway at any level. The low incidence of injury to the larynx is likely due to its protected position, by the mandible superiorly, the sternum inferiorly, and the spine posteriorly [12]. Blunt trauma is by far the most common mechanism of tracheal injury [13]. Presenting symptoms include dyspnea, dysphonia, hoarseness, stridor, neck pain, dysphagia, and hemoptysis. Physical exam findings may include tenderness over the larynx, subcutaneous emphysema, cyanosis, air escaping from a neck wound, large air leak after chest tube placement, or persistent pneumothorax despite chest tube placement. It is important to remember that the severity of symptoms does not always correspond with the extent of injury [14] A CT scan of the neck and chest is indicated in stable patients and can diagnose most laryngeal fractures and dislocations, as well as identify associated injuries. The esophagus is the most common site of associated injury in tracheobronchial trauma. There is also a high incidence of associated recurrent laryngeal nerve injury in patients with fracture of the cricoid cartilage because of the close proximity to the nerve. Injury to major vessels and the thyroid gland are also commonly seen. CT angiogram may also be indicated if there is suspicion for concomitant vascular injury. One series that investigated major vascular injuries associated with tracheobronchial injuries found that the carotid artery is the most commonly injured vessel [15]. Direct visualization with flexible fiberoptic laryngoscopy and bronchoscopy is very important in diagnosing airway injury at any level [14].

The American Academy of Otolaryngology-Head and Neck Surgery has accepted the Schaefer Classification System as the most useful since it allows the clinician to make treatment decisions based on severity of injury [16].

### Schaefer Classification System for determining the severity of laryngeal injuries ([16])

**Table:** 

Group	Severity of injury
1	Minor endolaryngeal hematomas or lacerations without detectable fractures
2	More severe edema, hematoma, minor mucosal disruption without exposed cartilage, or non-displaced fractures
3	Massive edema, large mucosal lacerations, exposed cartilage, displaced fractures or vocal cord immobility
4	Same as group 3, but more severe with disruption of anterior larynx, unstable fractures, two or more fracture lines, or severe mucosal injuries
5	Complete laryngotracheal separation

The management of every laryngeal injury includes an initial stabilization of airway Once the airway is secured, further diagnostic workup can commence.

### Evaluation and management based on the Schaefer Classification System ([16])

**Table:** 

Group	Evaluation	Management
1	Flexible fiber optic laryngoscopy	Generally medically managed and do not require surgical intervention. Helpful adjunctive medical treatments include steroids, antibiotics, humidification, voice rest
2	Direct laryngoscopy and esophagoscopy	Serial examinations, since the injuries may worsen over time. These injuries infrequently require a tracheostomy. Helpful adjunctive medical treatments as described above
3	Direct laryngoscopy and esophagoscopy performed in the operating room	Tracheostomy and surgical repair are often required. The following injuries of the larynx require surgical repair: disruption of the anterior commissure, major endolaryngeal lacerations, vocal cord tear, immobile vocal cord, cartilage exposure, displaced cartilage fractures
4	Direct laryngoscopy and esophagoscopy performed in the operating room	Tracheostomy is always required
		Surgical repair requires stent placement to maintain the integrity of the larynx
5	These patients present in severe respiratory distress and therefore endoscopic evaluation is delayed until an airway is secured	Disruption of the airway usually occurs above or below the cricoid cartilage, either at the cricothyroid membrane or cricotracheal junction. The airway is usually temporarily established using an endotracheal tube inserted through the neck directly into trachea distal to the site of transection. A complex laryngotracheal repair is then performed through a low cervical incision

## Conclusion

1. Stabilize the airway first. Immediate surgical airway procedure can be necessary in less familiar circumstances and environments. If possible, define landmarks before the procedure.
2. Defining anatomical zones is useful in penetrating injuries, although these do not guide diagnostic or therapeutic management completely.
3. In unstable patients, elective surgical exploration is recommended instead of extensive diagnostic work-up. Unstable patients still need immediate exploration, whereas all stable patients will first be assessed with clinical examination and CT angiography and fibreoptic laryngoscopy.

Thus the take home message is to consider all neck injuries an emergency and proceed with the diagnosis and management without delay.
